# Xylose Improves Antibiotic Activity of Chloramphenicol and Tetracycline against *K. pneumoniae* and *A. baumannii* in a Murine Model of Skin Infection

**DOI:** 10.1155/2018/3467219

**Published:** 2018-07-18

**Authors:** Alejandro A. Hidalgo, Ángel J. Arias, Juan A. Fuentes, Patricia García, Guido C. Mora, Nicolás A. Villagra

**Affiliations:** ^1^Laboratorio de Patogénesis Molecular y Antimicrobianos, Facultad de Medicina, Universidad Andres Bello, Santiago, Chile; ^2^Escuela de Química y Farmacia, Facultad de Medicina, Universidad Andres Bello, Santiago, Chile; ^3^Laboratorio de Genética y Patogénesis Bacteriana, Facultad de Ciencias de la Vida, Universidad Andres Bello, Santiago, Chile; ^4^Servicio de Laboratorios Clínicos Laboratorio de Microbiología, Escuela de Medicina, Pontificia Universidad Católica de Chile, Santiago, Chile

## Abstract

Increased resistance to antimicrobials in clinically important bacteria has been widely reported. The major mechanism causing multidrug resistance (MDR) is mediated by efflux pumps, proteins located in the cytoplasmic membrane to exclude antimicrobial drug. Some efflux pumps recognize and expel a variety of unrelated antimicrobial agents, while other efflux pumps can expel only one specific class of antibiotics. Previously, we have reported that xylose decreases the efflux-mediated antimicrobial resistance in *Salmonella typhimurium*, *Pseudomonas aeruginosa*, and *Acinetobacter baumannii in vitro*. In this work, we assessed the effectiveness of combining xylose with antibiotics to kill resistant *Acinetobacter baumannii* and *Klebsiella pneumoniae* in a murine model of skin infection. Skin infections were established by seeding 10^9^ bacteria onto eroded skin of mice. Mice treated with the antibiotic alone or with a mixture of glucose and antibiotics or xylose and antibiotics were compared to a control group that was infected but received no further treatment. We observed that the mixtures xylose-tetracycline and xylose-chloramphenicol produced a decrease of at least 10 times viable *Acinetobacter baumannii* and *Klebsiella pneumoniae* recovered from infected skin, compared with mice treated with the antibiotic alone. Our results show that xylose improves the antibiotic activity of tetracycline and chloramphenicol against efflux-mediated resistance *Acinetobacter baumannii* and *Klebsiella pneumoniae*, in a murine model of skin infection. We envision these combined formulations as an efficient treatment of skin infections with bacteria presenting efflux-mediated resistance, in both humans and animals.

## 1. Introduction

Skin infections are one of the most common infections [1]. Breaks in the skin, such as leg ulcers and surgical or traumatic wounds, constitute a perfect environment for infections by a broad range of bacteria [2]. Most skin infections are caused by Gram-positive bacteria, commonly *Staphylococcus aureus* and group A *β*-haemolytic *Streptococcus* [1]. However, Gram-negative bacteria such as *Acinetobacter baumannii*, *Pseudomonas aeruginosa*, and *Klebsiella pneumoniae* may also cause skin infections [2]. The incidence of skin infections has increased due to ageing of the general population, increased number of critically ill patients, increased number of immunocompromised patients, and recent emergence of multidrug-resistant pathogens [3]. Multidrug resistance (MDR) is defined as the resistant phenotype to antibiotics belonging to two or more classes of antibiotics and represents a serious problem in healthcare settings [4, 5]. Drug-resistant bacteria are responsible for more than 30,000 deaths per year in the UK and Europe, and it is estimated that 23,000 people in the United States die from pathogens that are not responsive to treatments with current antibiotic therapies [6].

Bacteria exhibit different strategies to resist antibiotics. One of the most important mechanisms, considered a major contributor to the emergence of MDR pathogens, is the antibiotic efflux achieved by efflux pumps [7]. Efflux pumps are proteins located in the inner membrane of Gram-negative bacteria and in the cytoplasmic membrane of Gram-positive bacteria [7]. The continuous onset of MDR in bacterial strains limits the clinical efficacy of most available antibiotics. Therefore, there is an urgent need to introduce novel antimicrobial molecules that may be active by themselves or potentiate current available antibiotics [8].

In a previous *in vitro* study, we found that xylose decreases the efflux-mediated antimicrobial resistance in *S. typhimurium*, *P. aeruginosa*, and *A. baumannii.* Although the mechanism behind sensitization remains elusive, it has been speculated that either competitions for limited space in the inner membrane or interference with the translocon systems may affect translocation of efflux pumps into membrane, thereby affecting efflux-mediated resistance [9]. Because the *in vitro* potentiation of actively expelled antimicrobials was fairly significant in the presence of xylose, we ought to find whether this potentiation can be reproduced *in vivo.* Therefore, in this work, we assessed the effectiveness of combining xylose with antibiotics *in vivo*. Our results show that xylose increases the antibiotic activity of tetracycline and chloramphenicol against efflux-dependent resistant *A. baumannii* and *K. pneumoniae,* in a model of skin infection in mice.

## 2. Materials and Methods

### 2.1. Bacterial Strains and Growth Conditions

Clinical strains of *A. baumannii* and *K. pneumoniae* were collected from different healthcare facilities throughout Santiago, Chile, and collected at Servicio de Laboratorios Clínicos, Escuela de Medicina, Pontificia Universidad Católica de Chile in Santiago, between 2014 and 2015. The *A. baumannii* strains were isolated from tracheal secretions from patients with respiratory infection. The *K. pneumoniae* strains were isolated from urine of patients with urinary infection. Strains were grown in LB broth at 37°C with aeration. Solid media (LB agar) included Bacto agar (15 g/L).

### 2.2. Antimicrobial Susceptibility Test

We used a modification of the disc diffusion assay previously described [9, 10]. Briefly, cultures were grown for 16  h in LB broth; bacteria were washed three times and resuspended in PBS. 10^6^ cells were spread on M9 plates supplemented with glucose or xylose (2 mg/mL) [9]. When required, the medium was supplemented with 12.5 *μ*M carbonyl cyanide-m-chlorophenylhydrazone (CCCP), an indirect efflux pumps inhibitor that acts by impairing the proton transport. Ten microliters of tetracycline 10 *μ*g/mL or chloramphenicol 20 *μ*g/mL were spotted in a sterile filter paper disc, placed on the center of the plate. The diameters of the inhibition haloes, obtained after an overnight culture at 37°C, were reported as the average of two measurements.

The MICs for each bacterial strain were determined by microdilution in liquid medium as recommended by the CLSI [11], with modifications. Briefly, bacteria were grown overnight in LB medium, washed 3 times with PBS, diluted in fresh M9 glucose medium or M9 xylose medium, and aliquoted (100 *μ*L) into the wells of sterile microtitre plates (10^6^ CFU/mL in each well). The lowest concentration of antimicrobial agents that inhibited growth (measured as the optical density at 640 nm) by at least 50%, relative to growth in the absence of antimicrobial agents, was defined as the MIC. When required, the medium was supplemented with 12.5 *μ*M CCCP.

### 2.3. Skin Infections

Female BALB/c mice (7–8 weeks old) were anesthetized with a mixture of 1 : 3 of xylazine 2% and ketamine 115 mg/mL intraperitoneally injected (1 *µ*L per g of weight). Followed by this, 1 cm^2^ of skin was shaved, on the back of each animal, to produce a slight irritation. The area was immediately infected with 20 *µ*L of a PBS-bacterial suspension containing 1 × 10^9^ CFU/mL of *A. baumannii* or *K. pneumoniae*. Littermate mice were distributed into four groups: the control group, which was only infected but received no further treatment; the antibiotic-treated group, which was treated topically with 20 *µ*L of antibiotics (tetracycline 2.5 *μ*g/mL when *A. baumannii* was used or chloramphenicol 2.5 *μ*g/mL when *K. pneumoniae* was used); and the glucose-antibiotic group and the xylose-antibiotic group, which were treated topically with a mixture of 20 *µ*L of 2.5 *μ*g/mL antibiotic (tetracycline or chloramphenicol) plus 20 *µ*L of sugar (glucose 2% or xylose 2%). The treatments started 4 h after infection and were repeated every 12 h. Three days after infection, mice were euthanized to dissect 1 cm^2^ of the infected areas, which were homogenized in 0.5 mL of PBS to obtain the CFU by plating onto LB agar. Bacterial recovered from the infected skin were confirmed as *A. baumannii* or *K. pneumoniae* by using API10s test. Experiments with animals were performed according to Protocol 012-2013 approved by the Bioethics Committee at Universidad Andrés Bello and in accordance with the NIH guide for the care and use of laboratory animal.

## 3. Results

### 3.1. The Presence of Xylose Decreased the Efflux-Pump-Mediated Antimicrobial Resistance in *A. baumannii* and *K. pneumoniae*

In a previous study, we determined that xylose, as the sole carbon source, decreases efflux-mediated resistance of Gram-negative bacteria to different antibiotics *in vitro* [9]. Since *A. baumannii* and *K. pneumoniae* are frequently resistant to tetracycline and chloramphenicol by mechanisms involving efflux pumps [12, 13], in this study we used these antibiotics to test the effect of xylose. In the* in vitro *experiments, we observed that 60% (9/15) of the *A. baumannii* strains tested increased their susceptibility to tetracycline when xylose was the sole carbon source (Supplementary Table S1). Similarly, 77% (10/13) of tested *K. pneumoniae* strains were more susceptible to chloramphenicol when xylose was the sole carbon source (Supplementary Table S1). To study how xylose may affect efflux-dependent resistance, we choose two strains of *A. baumannii* (34702 and 34280) and two strains of *K. pneumoniae* (28296 and 28341) to perform the *in vitro* and *in vivo* experiments. In Table 1, we show that *A. baumannii* 34702 and *K. pneumoniae* 28296 exhibited efflux-dependent resistance (EDR) to tetracycline or chloramphenicol, respectively, as assessed by the increased susceptibility in presence of the efflux pump inhibitor CCCP [9, 14]. It has been reported that resistance in *A. baumannii* to tetracycline is attained by expelling the drug mainly through the AedC, CraA, AdeB, AdeG, and AdeJ efflux pumps [12,15–17]. Similarly, in *K. pneumoniae*, the resistance to chloramphenicol is attained by expelling the drug mainly through the AcrB efflux pump [18]. By contrast, *A. baumannii* 34280 and *K. pneumoniae* 28341 showed efflux-independent resistance (EIR) to tetracycline or chloramphenicol, respectively, since the presence of CCCP exerted no effect in the growth inhibition haloes. Furthermore, the presence of xylose as the sole carbon source increased the antibiotic susceptibility only in the EDR strains (Table 1). To quantitatively confirm our results, we performed MIC assay for each bacterial strain. As observed in Table 1, the MIC for tetracycline in *A. baumannii* 34702 was 2 *μ*g/mL in presence of xylose, compared with 64 *μ*g/mL in the presence of glucose; therefore, the same strain was 32 times more susceptible to tetracycline in presence of xylose. In the same way, the results observed for *K. pneumoniae* 28296 showed that the MIC for chloramphenicol was 2 *μ*g/mL in the presence of xylose, compared with 32 *μ*g/mL in the presence of glucose; therefore, the same strain was 16 times more susceptible to chloramphenicol in the presence of xylose. These results confirm that both strains exhibited efflux-dependent resistance (EDR) to tetracycline or chloramphenicol, respectively.

As a control, we performed a growth curve for *A. baumannii *34702 and *K. pneumoniae* 28296 with glucose or xylose as the sole carbon source to determine the bacterial growth rate. The results in Figure S1 (in supplementary data) show that the growth rate of *A. baumannii* 34702 in xylose is slightly lower than that in the presence of glucose, as the sole carbon source (in logarithmic phase: *A. baumannii* + Glu: 0.18 h^−1^ and *A. baumannii* + Xyl: 0.17 h^−1^). A similar result was observed in *K. pneumoniae* 28296 (in logarithmic phase: *K. pneumoniae* + Glu: 0.19 h^−1^ and *K. pneumoniae* + Xyl: 0.16 h^−1^) (Figure S2 in supplementary data). Notwithstanding the lower growth rate in minimal medium plus xylose, the growth inhibition haloes were evident for both *A. baumannii and K. pneumoniae* in the presence of either tetracycline or chloramphenicol. In addition, the inhibition haloes measured after 12 h remained unmodified when measured after 24, 48, or 72 h (Tables S2 and S3, included in supplementary data). Thus, we conclude that xylose affects resistance to tetracycline or chloramphenicol independently from its effects on growth rates. These results strongly suggest that both strains exhibit efflux-dependent resistance (EDR) to tetracycline or chloramphenicol, respectively.

As expected, *A. baumannii* 34280 and *K. pneumoniae* 28341, both showing efflux-independent resistance (EIR) to tetracycline or chloramphenicol, presented no changes in the MIC, whether cultured in the presence of xylose or glucose as the sole carbon source (Table 1). In addition, the presence of CCCP exerted no effect on the MIC (Table 1, glucose versus glucose + CCCP). In our experiments, we used CCCP at concentrations ranging from 4 to 10 times less than used in previous reports [19, 20]. Furthermore, we assessed the growth of *A. baumannii* 34702 and *K. pneumoniae* 28296 in presence or absence of CCCP (12.5 *μ*M). The results in Figures S1 and S2 (supplementary data) show that CCCP does not affect the growth rate of *A. baumannii* 34702 (in logarithmic phase: *A. baumannii* + Glu: 0.18 h^−1^ and *A. baumannii* + Glu + CCCP: 0.33 h^−1^) or *K. pneumoniae* 28296 (in logarithmic phase: *K. pneumoniae* + Glu: 0.19 h^−*1*^ and *K. pneumoniae* + Glu + CCCP: 0.14 h^−1^).

Altogether, these *in vitro* experiments show that xylose potentiated the antibiotic activity of tetracycline and chloramphenicol in EDR strains of *A. baumannii* and *K. pneumoniae*.

### 3.2. Xylose Potentiates the Antibiotic Activity of Tetracycline and Chloramphenicol against EDR Strains of *A. baumannii* and *K. pneumoniae* in a Murine Model of Skin Infection

**Figure 1:**
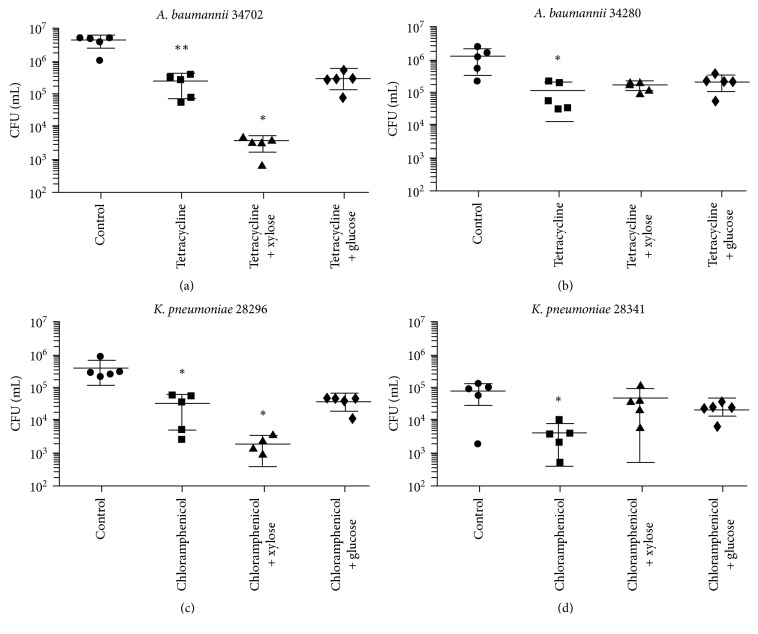
Xylose potentiates antibiotic activity of efflux-dependent resistant bacteria in a murine model of skin infection. The ability of xylose to increase the susceptibility of clinical *A. baumannii* and *K. pneumomiae* strains to tetracycline and chloramphenicol, respectively, was determined *in vivo*. Bacteria were used to infect skin lesion in mice, prior to treating mice with the antibiotic alone or with a mixture of the antibiotic and xylose. As control, a group of mice was treated with antibiotic and glucose, while another was left untreated. We tested the EDR strain *A. baumannii* 34702 (a) and the EIR strain *A. baumannii* 34280 (b) with tetracycline; and the EDR strain *K. pneumomiae* 28296 (c) and the EIR strain *K. pneumomiae* 28341 (d) with chloramphenicol. Results are expressed as CFU/mL of homogenized tissue. Experiments were repeated at least 3 times. ^*∗*^ *p* < 0.05 and ^*∗∗*^ *p* < 0.01 according to Student's *t*-test.

To determine whether xylose can potentiate antibiotic activity, not only *in vitro* but also *in vivo*, a murine model of skin infection was chosen using the same strains and antibiotics described in Table 1. Skin infections were established by seeding *A. baumannii* or *K. pneumoniae* on the skin of mice, previously shaved to generate lesions, as previously described in method sections and in references [18]. Bacteria recovered from the infected skin and treated with the antibiotics or with a mixture of glucose and antibiotics or xylose and antibiotics were compared with a control group that was infected but received no further treatment. As shown in Figure 1(a), we recovered 10 times less CFU of *A. baumannii* 34702 (EDR strain) from tetracycline-treated mice than from the untreated group. In addition, we observed that the mixture of xylose and tetracycline was the most efficient treatment, decreasing the recovery of *A. baumannii* 34702 100 times compared with the untreated control mice and 10 times compared with the tetracycline-treated mice. Control group of infected mice treated with xylose alone and mice treated with a mixture of glucose and tetracycline showed no differences in the bacterial count, compared to untreated control group and antibiotic-treated group, respectively. Importantly, the addition of xylose did not improve the effect of tetracycline in mice infected with *A. baumannii* 34280, an EIR strain, as expected from our *in vitro* assays (Figure 1(b)).

We obtained similar results with *K. pneumoniae*, where xylose clearly improved the effect of chloramphenicol only in *K. pneumoniae* 28296 (300 times compared to the recovered CFU from untreated mice and 30 times compared to the antibiotic-treated group), an EDR strain, but not in *K. pneumoniae* 28341, an EIR strain (Figures 1(c) and 1(d)). A control group of infected mice treated with xylose alone and mice treated with a mixture of glucose and chloramphenicol showed no differences in the bacterial count. Again, these results are consistent with those obtained *in vitro* (Table 1).

As a control, we grew *A. baumannii* 34702 and *K. pneumoniae* 28296 in the presence of different concentrations of xylose. Our observations did not show impaired growth or antibacterial effect of this sugar alone against bacteria (Table S4 in supplementary data).

## 4. Discussion

The data presented show that xylose can be used to potentiate the antibiotic activity of tetracycline and chloramphenicol *in vivo* in strains presenting EDR. For this study, we have chosen tetracycline and chloramphenicol, antibiotics which resistance is commonly achieved through efflux pumps. The antibiotic tetracycline is still used against both Gram-positive and Gram-negative bacteria. Although tetracycline is not the first choice against skin infections because it can cause phototoxic reactions [21], it is useful in several other types of infections, including rickettsial, chlamydial and periodontal infections, and atypical pneumonias [22]. Considering that tetracycline resistance easily arises because of horizontal gene transfer encoding efflux systems, the results described in this manuscript may have a major impact on the treatment of tetracycline-resistant bacteria.

Chloramphenicol presents excellent *in vitro* activity against most anaerobes, which are a major cause of skin infections [23]. Therefore, the evidence presented here suggests that adding xylose to antibiotics from different families is useful for increasing the antimicrobial effect of antibiotics that show prevalence for resistance through active efflux. In a previous work, we hypothesized that it is possible to alter the efflux pump-mediated antibiotic resistance by overproducing unrelated inner membrane proteins, such as carbohydrate permeases. An easy way to increase the production of permeases is to culture bacteria with a non-PTS carbohydrate as the sole carbon source, such as xylose. Indeed, xylose was the best non-PTS sugar at potentiating antibiotic effects, compared to galactose or arabinose [9]. In addition, xylose was chosen because it is apparently innocuous, and its pharmacokinetic/pharmacodynamics parameters have been extensively studied. Indeed, xylose is administered systemically at high doses to assess intestinal absorption in human.

In a next step, we will incorporate various excipients to the mixture of antibiotic and xylose to enhance the effect as part of a topic formulation. Importantly, and beside our predictions, wound exudates, which may contain glucose, a sugar that exerts catabolic repression over xylose metabolism (Table S5 in supplementary data) apparently did not inhibit the *in vivo* effects of xylose regarding antibiotic sensitization.

## 5. Conclusion

In summary, the data we presented here demonstrate that xylose potentiates the *in vivo* antibiotics effect of tetracycline and chloramphenicol in bacteria that actively expel these antibiotics. Such effect was previously demonstrated *in vitro* for tetracycline and chloramphenicol, both actively expelled antibiotics. In addition, we show evidence that xylose is effective in potentiating antibiotic activity of tetracycline and chloramphenicol besides glucose present on wound exudates. These results are not only a proof of principles but also ground to expand the use of xylose with two purposes: uncovering EDR mechanisms and potentiating susceptibility of bacteria showing EDR, to antibiotics tested in this study and others.

## Figures and Tables

**Table 1:** Susceptibility profile of *A. baumannii* and *K. pneumoniae* strains used in a model of skin infection in mice.

Strains	Resistance	Antibiotics	Inhibition halo (mm)			MIC (*μ*g/mL)		
			Glu	Xyl	Glu + CCCP	Glu	Xyl	Glu + CCCP
*A. baumannii* 34702	EDR	Tet	45	50	52	64	2	32
*A. baumannii* 34280	EIR	Tet	45	45	44	64	64	64
*K. pneumoniae* 28296	EDR	Cam	39	49	48	32	2	16
*K. pneumoniae* 28341	EIR	Cam	13	15	15	8	8	8

## Data Availability

All data generated during or analyzed during this study are included in this published article and supplementary figures and tables.

## Abbreviations

MDR: Multidrug resistant
CCCP: Carbonyl cyanide-m-chlorophenylhydrazone
MIC: Minimal inhibitory concentration
M9: Minimal medium 9
CFU: Colony formation unit
EDR: Efflux-dependent resistance
IDR: Efflux-independent resistance.

## Supplementary Materials

Supplementary Materials Table S1: *A. baumannii* and *K. pneumoniae* strains used in this study, and their susceptibility to tetracycline and chloramphenicol in the presence of glucose or xylose. Table S2: inhibition haloes of *A. baumannii* 34702 and *K. pneumoniae* 28296 measured in millimeter after 12 hours of incubation. Table S3: inhibition haloes of *A. baumannii* 34702 and *K. pneumoniae* 28296 measured in millimeter after 72 hours of incubation. Table S4: susceptibility profile of *A. baumannii* 34702 and *K. pneumoniae* 28296 strains with different concentrations of xylose. Table S5: susceptibility profile of *A. baumannii* and *K. pneumoniae* strains in the presence of glucose, xylose, and a mixture of glucose and xylose. Figure S1: growth curves for *A. baumannii* 34702 in the presence of glucose or xylose, as the sole carbón source, and glucose plus CCCP. Figure S2: growth curves for *K. pneumoniae* 28296 in the presence of glucose or xylose, as the sole carbón source, and glucose plus CCCP.
