# Supplementary material for: Xylose Improves Antibiotic Activity of Chloramphenicol and Tetracycline against K. pneumoniae and A. baumannii in a Murine Model of Skin Infection
Source: Can J Infect Dis Med Microbiol. 2018 Jul 18;2018:3467219. doi: 10.1155/2018/3467219 (PMC6079543; doi:10.1155/2018/3467219)
Supplement: Supplementary Materials — Table S1: A. baumannii and K. pneumoniae strains used in this study, and their susceptibility to tetracycline and chloramphenicol in the presence of glucose or xylose. Table S2: inhibition haloes of A. baumannii 34702 and K. pneumoniae 28296 measured in millimeter after 12 hours of incubation. Table S3: inhibition haloes of A. baumannii 34702 and K. pneumoniae 28296 measured in millimeter after 72 hours of incubation. Table S4: susceptibility profile of A. baumannii 34702 and K. pneumoniae 28296 strains with different concentrations of xylose. Table S5: susceptibility profile of A. baumannii and K. pneumoniae strains in the presence of glucose, xylose, and a mixture of glucose and xylose. Figure S1: growth curves for A. baumannii 34702 in the presence of glucose or xylose, as the sole carbón source, and glucose plus CCCP. Figure S2: growth curves for K. pneumoniae 28296 in the presence of glucose or xylose, as the sole carbón source, and glucose plus CCCP. [file 3467219.f1.pdf]

Supplementary Data for:

Xylose improves antibiotic activity of chloramphenicol and tetracycline against *K. pneumoniae* and *A. baumannii* in a murine model of skin infection

Alejandro A. Hidalgo, Ángel J. Arias, Juan A. Fuentes, Patricia García, Guido C. Mora, Nicolás A. Villagra

Table S1: *A. baumannii* and *K. pneumoniae* strains used in this study and their susceptibility to tetracycline and chloramphenicol in the presence of glucose or xylose, as the sole carbon source, and the presence of the efflux pump inhibitor CCCP.

Strains	Resistance mechanism	Antibiotics	Inhibition Halo (mm)		
			Glu	Xyl	Glu+CCCP
A.baumannii 34702	EDR	Tet	45	50	52
A.baumannii 34724	EDR	Tet	38	43	43
A.baumannii 34938	EDR	Tet	38	44	43
A.baumannii 18628	EDR	Tet	37	43	44
A.baumannii 19328	EDR	Tet	38	42	43
A.baumannii 18624	EDR	Tet	36	42	42
A.baumannii 18395	EDR	Tet	38	43	43
A.baumannii 18698	EDR	Tet	37	44	44
A.baumannii 18684	EDR	Tet	37	42	43
A.baumannii 34280	EIR	Tet	45	45	44
A.baumannii 34562	EIR	Tet	38	38	38
A.baumannii 18394	EIR	Tet	37	37	37
A.baumannii 19381	EIR	Tet	36	37	36
A.baumannii 19075	EIR	Tet	38	38	38
A.baumannii 18775	EIR	Tet	37	38	37
K. pneumoniae 28296	EDR	Cam	39	49	48
K. pneumoniae 27935	EDR	Cam	30	37	36
K. pneumoniae 28295	EDR	Cam	38	47	47
K. pneumoniae 27698	EDR	Cam	38	47	46
K. pneumoniae 28115	EDR	Cam	29	38	38
K. pneumoniae 28532	EDR	Cam	31	38	37
K. pneumoniae 28106	EDR	Cam	29	36	37
K. pneumoniae 28558	EDR	Cam	28	37	36
K. pneumoniae 28552	EDR	Cam	26	35	37
K. pneumoniae 28155	EDR	Cam	28	38	38
K. pneumoniae 28341	EIR	Cam	13	15	15
K. pneumoniae 1705	EIR	Cam	15	15	15
K. pneumoniae 27665	EIR	Cam	13	13	14

EDR: Efflux-Dependent Resistance; EIR: Efflux-Independent Resistance; Tet, tetracycline; Cam, chloramphenicol.

Table S2. Inhibition haloes (measured in mm after 12 h of incubation)

Bacteria	Antibiotics	Inhibition Halo (mm)	
		Glucose	Xylose
A. baumannii 34702	Tetracycline	45	50
K. pneumoniae 28296	Chloramphenicol	39	48

Table S3. Inhibition haloes (measured in mm after 72 h of incubation)

Bacteria	Antibiotics	Inhibition Halo (mm)	
		Glucose	Xylose
A. baumannii 34702	Tetracycline	45	50
K. pneumoniae 28296	Chloramphenicol	39	48

Table S4: Susceptibility profile of *A. baumannii* and *K. pneumoniae* strains with different concentration of xylose.

Bacteria	Antibiotics	Inhibition Halo (mm)		
		Glucose	Xylose 0,2%	Xylose 2%
A. baumannii 34702	Tetracycline	45	50	51
K. pneumoniae 28296	Chloramphenicol	39	49	49

Table S5: Susceptibility profile of *A. baumannii* and *K. pneumoniae* strains in the presence of glucose, xylose and a mixture of glucose and xylose.

Bacteria	Antibiotics	Inhibition Halo (mm)		
		Glucose	Xylose	Glucose + Xylose
A. baumannii 34702	Tetracycline	45	50	44
K. pneumoniae 28296	Chloramphenicol	39	49	39

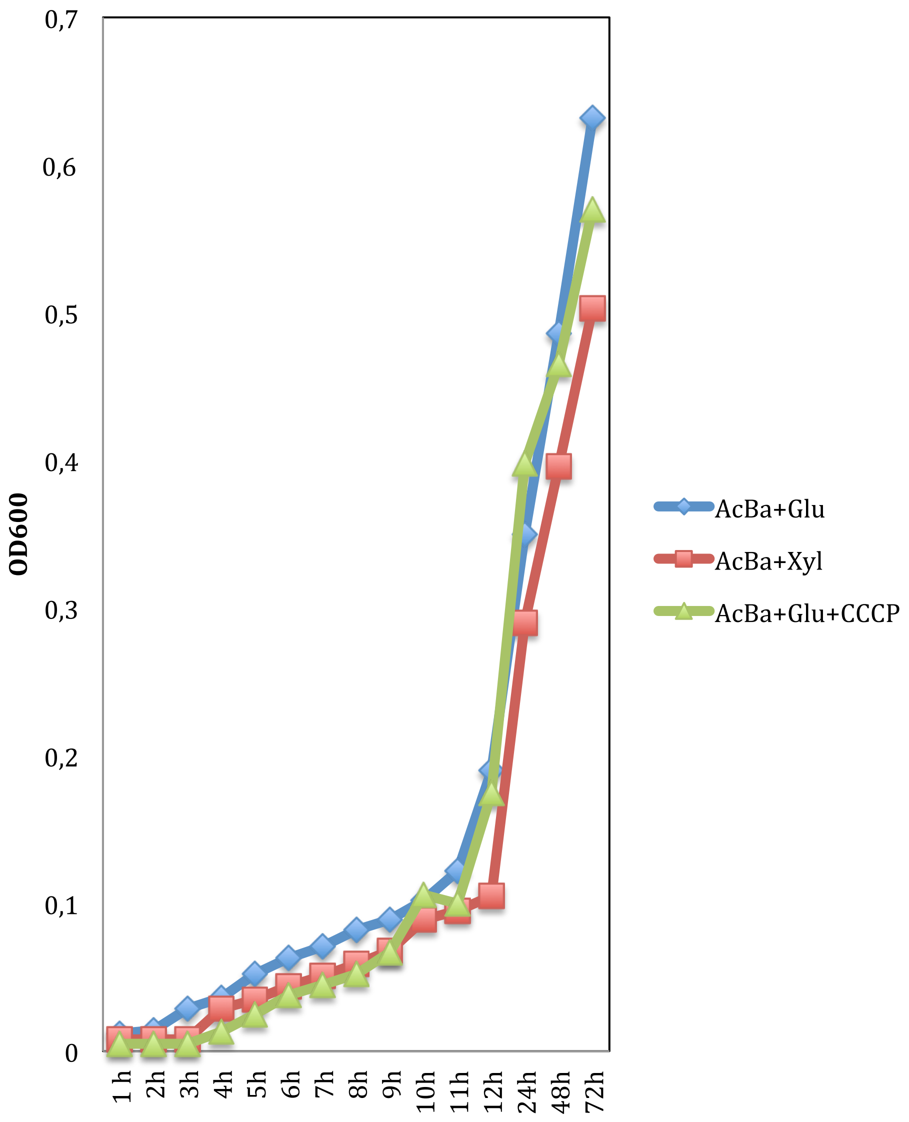

Figure S1: Growth curves for *A. baumannii* 34702 (AcBa) in the presence of glucose or xylose, as the sole carbon sources, and glucose plus CCCP.

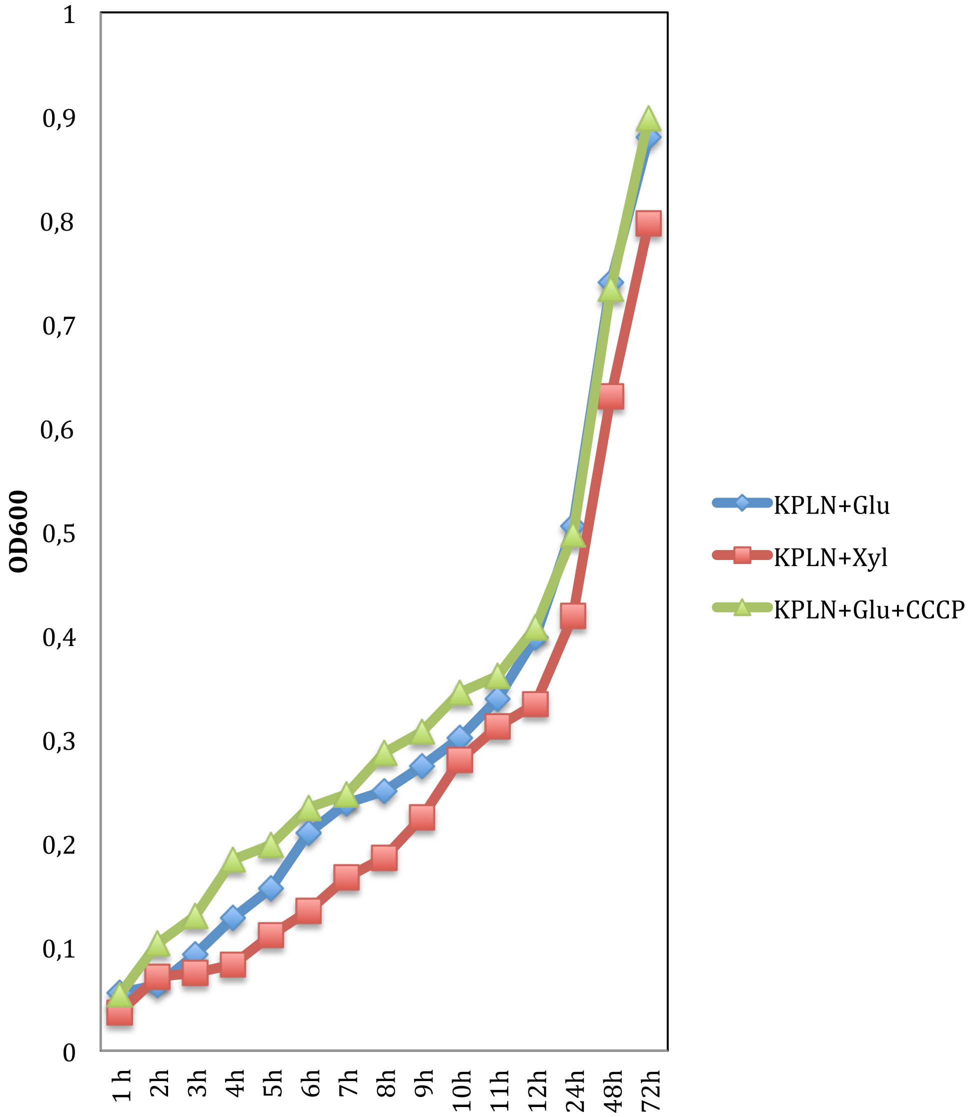

Figure S2: Growth curves for *K. pneumoniae* 28296 (KPLN) in the presence of glucose or xylose, as the sole carbon source, and glucose plus CCCP.

Highlights

1. Xylose decreases efflux-mediated resistance in multiresistant bacteria *in vitro*
2. Xylose improves antibiotics activity against multiresistant bacteria *in vivo*
3. Xylose + antibiotic formulations work efficiently to treat skin infections

Conflict of interest

None to declare
